# Fully automated robotic ultrasound gallbladder imaging with subcostal scanning

**DOI:** 10.1038/s41598-025-31892-4

**Published:** 2025-12-10

**Authors:** Koudai Okuzaki, Norihiro Koizumi, Shota Takahashi, Ryota Masuzaki, Naoki Matsumoto, Masahiro Ogawa, Kiyoshi Yoshinaka, Ryosuke Tsumura

**Affiliations:** 1 Health and Medical Research Institute, National Institute of Advanced Industrial Science and Technology, Tsukuba, Japan; 2 Graduate School of Informatics and Engineering, University of Electro- Communications, Tokyo, Japan; 3 Department of Gastroenterology, Nihon University Hospital, Tokyo, Japan

**Keywords:** Robotic ultrasound, Remote healthcare, Gallbladder imaging, Medical robot, Engineering, Health care, Medical research

## Abstract

To overcome the operator-dependence of ultrasound (US) imaging, robotic US systems have been widely developed for fully automated scanning. In this study, toward fully automated screening of gallbladder in abdominal US examinations, we propose a scan path planning method and a probe posture control strategy using our robotic US system. The path planning method uses an RGB-D camera to capture changes in the patient’s chest height position due to breathing. This allows the boundary line of the subcostal, which is an important landmark for the gallbladder scan, to be identified, and the scanning path for the robot is automatically generated. Regarding the probe posture control strategy, for proper contact between the probe and the body surface, we combine probe posture control with body surface normal estimation using the RGB-D camera before scanning and deep learning-based visual servoing to avoid shadows during scanning. These were implemented in our robotic US system and feasibility experiments were conducted on six healthy subjects. When the path planning method and the posture control strategy proposed in this study were applied, the detection rate of the gallbladder was 78.3%. These results demonstrate the possibility of fully automated gallbladder screening using the proposed system.

**Supplementary Information:**

The online version contains supplementary material available at 10.1038/s41598-025-31892-4.

## Introduction

 Ultrasound (US) imaging is one of the indispensable modalities in today’s medical practice because it is noninvasive, exposure-free, and inexpensive compared to CT and MRI^1^. One disadvantage of ultrasonography, however, is that it is highly operator-dependent. In order to obtain diagnostic US images that show the organs to be observed and are free of shadows and other obstructions that interfere with the observation, it is essential to have the skills of an experienced physician, such as knowledge of anatomy, delicate adjustment of probe position, and recognition of the relationship between the probe position and the US image^2^. In addition, because physicians perform examinations for long periods of time in unnatural postures, they develop musculoskeletal disorders at a high incidence rate of 63–98.7%^3,4^. To overcome this disadvantage, automation of US imaging with a robot-assisted technologies has been widely studied. Among them, there are still few studies that have realized fully-automated robotic US imaging, which can perform the entire process from path planning to image acquisition automatically^5^.

US imaging has a wide range of applications, such as fetal monitoring, diagnosis of cancers of the breast, thyroid gland, and other organs, and diagnosis of arteriosclerosis and thrombosis in carotid arteries and extremity blood vessels. Among these, abdominal US screening is a major application because it enables comprehensive observation of blood vessels and organs in the abdominal region, identification of the causes of abdominal pain and bloating, and detection of kidney stones, liver disease, tumors, abdominal aortic aneurysms, and many other diseases. The organs observed on abdominal US are primarily the gallbladder, kidney, liver, pancreas, and spleen. Among these, the gallbladder is the organ that can be diagnosed most accurately by US imaging rather than CT or MRI^6^. US imaging of the gallbladder is commonly used to evaluate gallbladder health, detect the presence of gallstones, polyps, inflammation, or blockages in the bile ducts, and then a key method in diagnosing conditions that may cause abdominal pain or digestive issues.

While there are many reports of robotic US imaging of the breast, extremities, thyroid, and fetus, there are few studies on the abdomen and only a few on the liver and abdominal aorta^7,8^. In particular, no studies specific to gallbladder imaging have been reported. The gallbladder is located beneath the liver, and US imaging of the gallbladder requires the use of the ribs as landmarks for accurate placement of the US probe on the right subcostal border^9^. In addition, the subcostal area is the boundary between the rib area and the abdomen, where the body surface is uneven and tissue stiffness varies^10^. Therefore, it is assumed that shadow noise often occurs in US images when there is insufficient contact between the US probe and the body surface, for example, when the US probe is only partially in contact due to the unevenness of the body surface when finding the gallbladder. The shadow noise may mask the gallbladder and surrounding tissue in the US image.

In summary, the accurate scanning position estimation along the boundary of the right subcostal and the proper US probe contact with the body surface are important toward fully automated US imaging of the gallbladder.

### Related works

One of the key technical challenges for fully automating the robotic US imaging is a scan path planning, which involves autonomously planning where to place the US probe on the body surface before starting the scanning. Victorova et al. started the scanning process by manually positioning the probe in its initial position, and then scanned the probe automatically along the spine^11^. Shida et al. performed US screening of the heart by scanning the area in its entirety, starting from the initial position where the probe was already in place^12^. Ma et al. manually attached markers to the surface of the human body, and the robot system read the markers and used them as scanning points^13^. In these studies, the initial position of the US probe was determined manually or with additional markers, and the scanning path was also specified manually or deterministically^11–13^. Tsumura et al. used statistical information about the geometric position of body landmarks to define the landing point of the auscultation robot^14^. Jiyong et al. defined the area for breast scanning based on statistical information^15^. These studies are based on body shape statistics but cannot sufficiently adapt to individual differences. Hennersperger et al. obtained pre-MRI images, detected the abdominal aorta in the MRI images, and generated the scanning path of the ultrasound probe along the abdominal aorta by registering it with the body surface information. This method requires additional modalities such as MRI, which detracts from the ease of US^8^. Deng et al. generated a scanning path by using imitation learning with US examination technique data^16^. Bi et al. constructed a virtual environment to simulate US examinations from CT data and performed reinforcement learning to generate scanning paths for intercostal scanning of the liver^17^. These deep learning-based methods require a huge amount of data and many trials for training. In short, most previous studies required additional human intervention or had problems adapting to individual differences. Several researches proposed to automatically generate scanning paths adapting to individual differences, but they required other medical imaging modalities or large dataset for the training of deep learning.

Another challenge for fully automating the robotic US imaging is avoiding shadow noise caused by insufficient contact of the US probe with the body surface, and several studies have addressed the issue. As a pre-scan process, there are many methods for controlling the direction of the probe in the normal direction of the body surface, such as depth camera-based^13,18,19^, distance sensor-based^20^, and force sensor-based^21^ methods. As a process during scanning, Huang et al. attached force sensors to both ends of the probe contact surface and corrected the probe rotation based on the force difference between the left and right force sensors to reduce shadows^18^. Jiang et al. reduced shading by correcting the rotation of the probe using visual servoing with an US confidence map^22^.

In scanning of the gallbladder, shadows can also occur due to the unevenness and changes in the stiffness of the body surface near the boundary of the subcostal, so it is important to control the rotational angle of the US probe to avoid the shadow noise. However, no study has focused on avoiding the shadows in scanning the gallbladder.

### Objectives and contributions

**Fig. 1:**
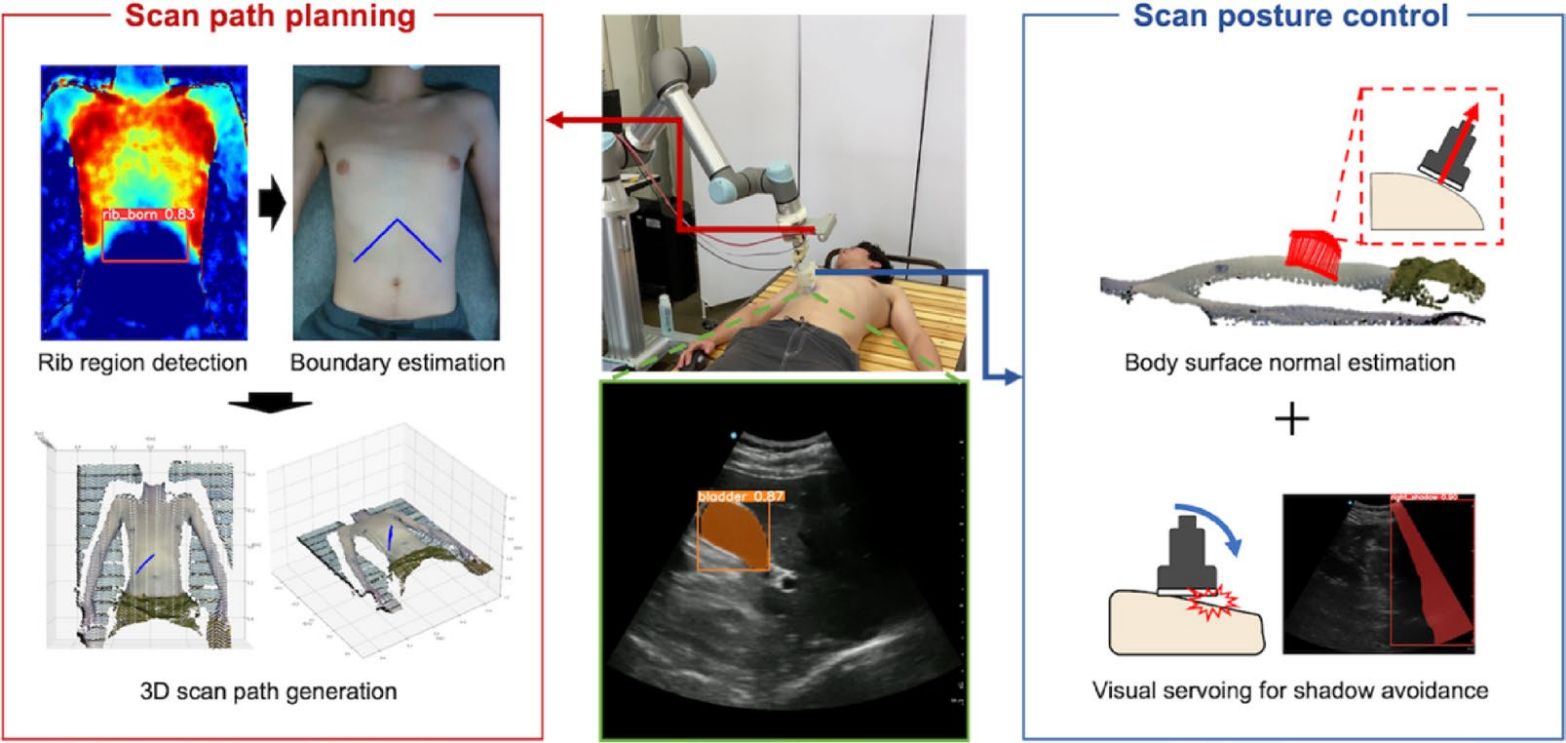
Proposed system overview consisted of scan path planning and scan posture control strategy.

To address the limitation of the related studies, this study aims to develop a robotic US imaging system to automate the screening of the gallbladder. The proposed system consists of two major elements: First, as a scanning path planning element, we estimate the rib region and the right subcostal arch scanning path by quantifying the displacement of the body surface during patient’s inspiration and expiration using an RGB-D camera. The proof-of-concept for the estimation of the rib region has been presented in a previous study^23^. On the other hand, the previous study did not implement the estimation method for the robotic US imaging system, nor did it verify its feasibility for clinical application. In this study, we attempt a pre-clinical application for the scan path planning of gallbladder in the right subcostal area are with the proposed estimation method. Next, during scanning along the estimated path, the US probe posture is controlled in two steps: first, it is oriented perpendicular to the body surface based on the pre-scan information; second, its rotational angle is refined through a visual-servoing approach to avoid acoustic shadows. Each of these elements is integrated as show in Fig. 1 and preclinically tested on healthy subjects. We believe that this is the first study focusing on the robotic US imaging for fully automated screening of the gallbladder and to conduct it on human trials.

## Method

### System overview

**Fig. 2:**
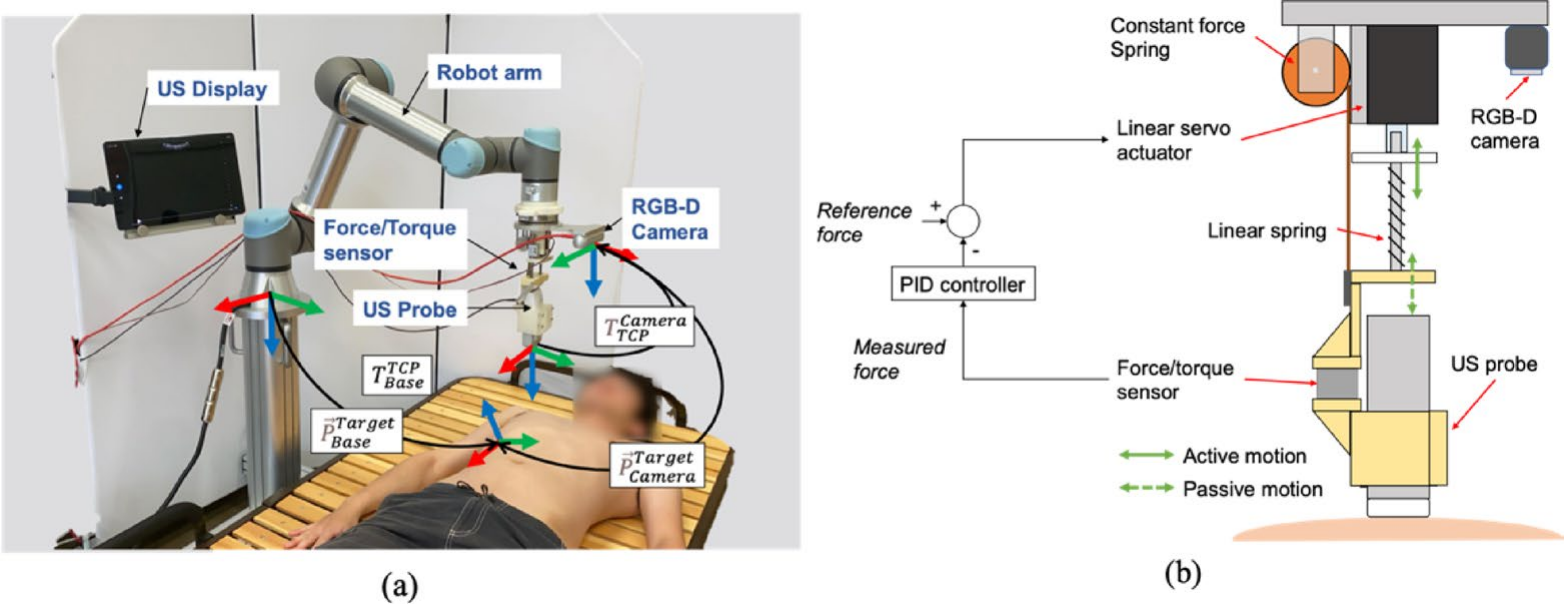
(**a**) Robotic US system overview and system coordinates and (**b**) the passive-actuated contact force end-effector.

The proposed US robot system consists of a 6-Degree-of-Freedom (DOF) cooperative robotic arm (UR5e, Universal Robots, Denmark), a wireless US probe (Vscan Air CL, GE Healthcare, USA), an end-effector that passively holds the US probe with a constant contact force, and a RGB-D camera (RealSense D435i, Intel, USA) to acquire 3D information on the body surface. The end-effector uses a passive-actuated mechanism and consists of a linear actuator (MightyZAP, IR ROBOT, Korea), a linear spring, a constant load spring, and a force sensor (ATI Nano 17, ATI Industrial, USA) to hold the US probe under maintaining arbitrary contact force constantly^24^ (see Fig. 2b). By controlling the contact force through the elastic elements, stable control is achieved against unevenness of the body surface and force variations in response to body motion. In addition to the safety contact, this system automatically stops when detecting excessive force above 20 N against the robot arm.

The RGB-D camera fixed on the end-effector is used to acquire 3D point cloud information of the body surface displacement during deep breathing, and the rib region and right subcostal arch scanning path are estimated. At the same time as scanning path planning, the normal vector to the body surface is calculated from the acquired 3D point cloud. Thereby the 6-DOF scanning position information in which the US probe touches perpendicularly to the body surface can be obtained. In order to accurately position the US probe held by the robotic arm in relation to the estimated path, we performed a hand-eye calibration in advance. The US probe was wirelessly connected to a tablet PC, which displayed US images. This US image is sent to the main control PC and is used for visual servoing to avoid the shadows.

### Path planning

**Fig. 3:**
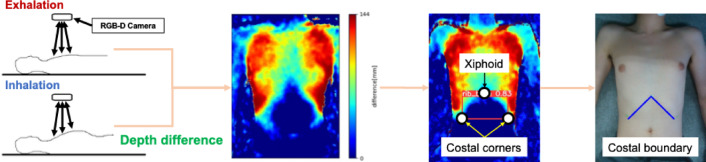
Estimation process of rib costal area and boundary.

To automatically acquire the US images of the gallbladder, it is necessary to move the US probe along the right subcostal boundary. To do so, the scanning path on the body surface to sweep under the right subcostal boundary needs to be estimated and converted to the robot’s coordinate system. First, the rib region, a landmark for gallbladder detection, is identified using the RGB-D camera. Although several methods of rib region extraction have been proposed^25,26^, we use our previously proposed method that allows non-contact estimation of the rib regions of various body shapes by observing the body surface displacement of the chest during breathing^23^. The chest during normal breathing and deep breathing are each captured with the RGB-D camera, and depth images including chest height information are acquired. By taking the difference between these two depth images and generating a depth difference image, the area of largest displacement during deep breathing, i.e., the rib region, is identified (see Fig. 3). From the rib regions identified by the depth difference image, the position of the xiphoid and the lower left and right ends of the identified rib region are detected using the object detection algorithm YOLOv5^27^. Details of these processes are described in the previous study^23^. The position of the right subcostal boundary is estimated by connecting the detected xiphoid position and the lower right end position of the rib (see Fig. 3).

The line indicating the subcostal boundary estimated on the 2D image is transformed onto the 3D point cloud acquired by the RGB-D camera, and the 3D position of the scanning position in the camera coordinate system vector $\:{\overrightarrow{P}}_{Camera}^{Target}$ are obtained. These coordinates are transformed using the transformation matrix $\:{\boldsymbol{T}}_{TCP}^{Camera}$ from the Tool Center Point (TCP) of the robot obtained from the hand-eye calibration to the origin of the camera coordinate system. Furthermore, the $\:{\boldsymbol{T}}_{Base}^{TCP}$ output from the robot’s controller is used to transform to the robot’s base coordinate system. These transformations are expressed by the following equations. $\:{\overrightarrow{P}}_{Base}^{Target}$ to be input to the robot can be determined (see Fig. 2 a).

1 $$\begin{array}{*{20}c} {\vec{P}_{{Base}}^{{Target}} = \user2{T}_{{Base}}^{{TCP}} \user2{T}_{{TCP}}^{{Camera}} \vec{P}_{{Camera}}^{{Target}} } \\ \end{array}$$

### Posture control

When applying the US probe to the estimated scanning path, the posture control of the US probe is important to avoid the shadows caused by the insufficient contact, i.e. the partial contact of the US probe. The void between the body surface and the US probe causes US attenuation and produces shadows on the US image. Since the void can be reduced by placing the US probe perpendicular to the body surface, the posture control is performed to align the vertical axis of the US probe with the normal vector of the body surface. To estimate the normal vector of the body surface, the following equation is calculated using principal component analysis (PCA) from a set of point cloud near the scanning path captured with the RGB-D camera. The covariance matrix $\:C$ can be represented by $\:k$ neighborhood points $\:{p}_{i}$ and their center of mass $\:\stackrel{-}{p}$.

$${C = \frac{1}{k}\sum\limits_{{i = 1}}^{k} { \cdot (p_{i} - \mathop p\limits^{ - } ) \cdot \:\left( {p_{i} - \mathop p\limits^{ - } } \right)^{T} } }$$

2 $$\begin{array}{*{20}c} {C \cdot \overrightarrow {{v_{j} }} = \lambda _{j} \cdot \overrightarrow {{v_{j} }} ,\quad ~~j \in \left\{ {0,1,2} \right\}} \\ \end{array}$$

**Fig. 4:**
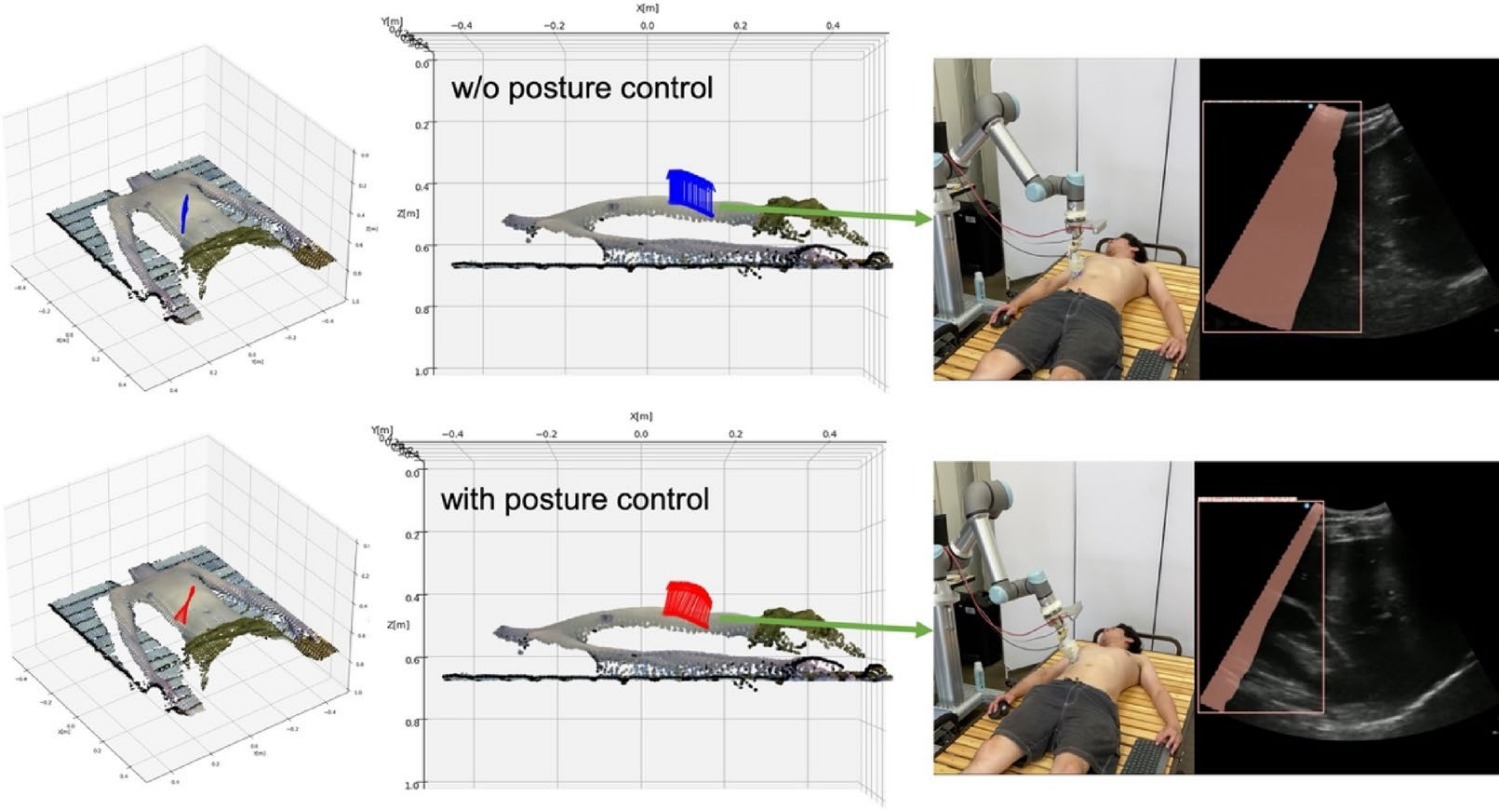
Comparison with and w/o posture control perpendicular to the body surface.

The eigenvalue $\:{\lambda\:}_{j}$ is the minimum of the eigenvalues $\:\overrightarrow{{v}_{j}}$. Let this be the normal $\:\overrightarrow{{n}_{i}}$. by satisfying the following equation,

3 $${\overrightarrow {{n_{i} }} \cdot \left( {v_{{viewpoint}} - p_{i} } \right)> 0}$$

where$$\:\:{v}_{viewpoint}$$ are the coordinates of the camera coordinate origin. The posture angle obtained as a normal vector $$\overset{\lower0.5em\hbox{$\smash{\scriptscriptstyle\rightharpoonup}$}}{{n_{i} }}$$ is also converted to the robot coordinate system and used as an input. In addition, scanning in which the US probe posture is fixed perpendicular to the floor surface is used as a comparison, without applying the above posture control (see Fig. 4).

### Visual servoing

**Fig. 5:**
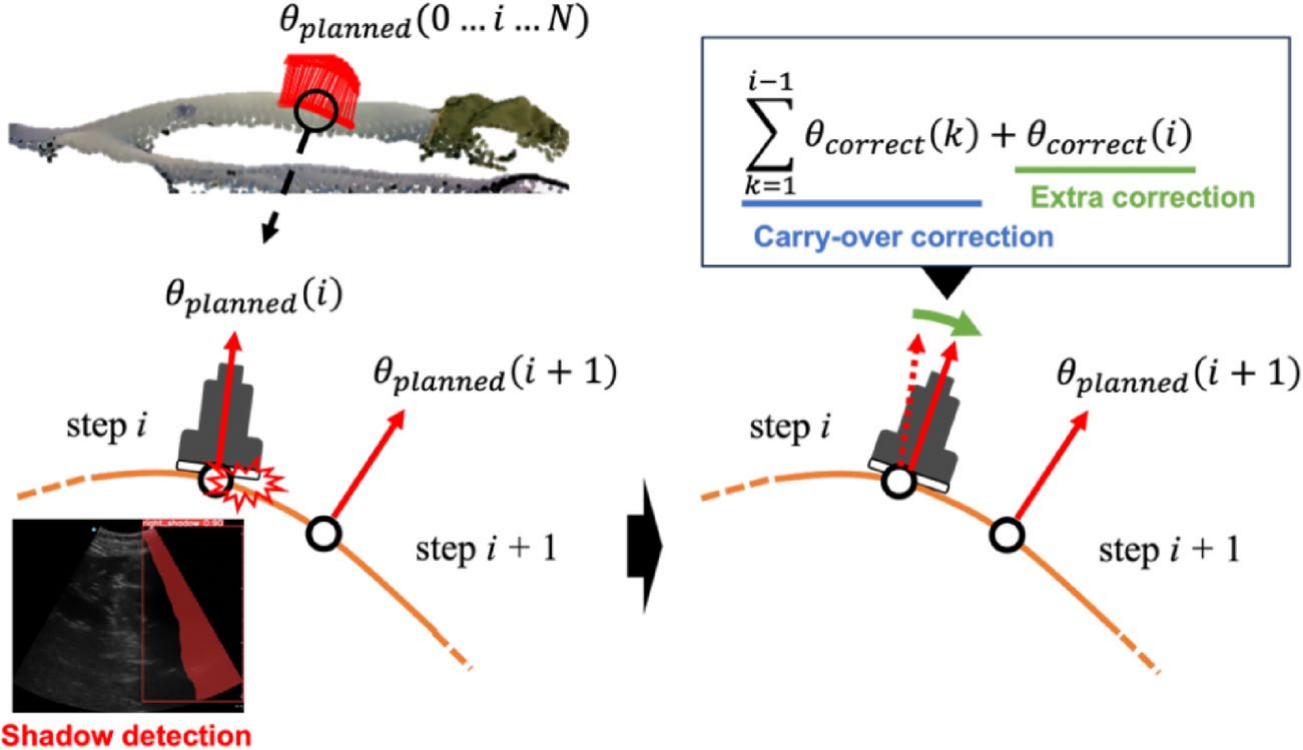
Overview of posture control method for shadow avoidance with visual servoing.

Controlling the posture angle so that the US probe is perpendicular to the body surface is expected to reduce the shadow occurrence. However, depending on the body type, there may be local irregularities on the body surface near the subcostal boundary, and the shadows may not be adequately avoided by simply placing the US probe perpendicular to the body surface. Existing methods to avoid shadows include placing a force sensor on the imaging surface of the probe^18^ and correcting the posture from the moment applied to the US probe^11^. In this study, in order to place emphasis on improving image quality, we applied a visual servo-based approach. First, the shadows are detected from acquired US images. There are several methods for detecting the shadows using US image confidence maps^22^ and luminance values^28^. While, in terms of the stability of visual servo control, this study applied an instance segmentation with YOLOv8^29^, which has both high real-time performance and high detection accuracy, to detect the shadows. It detects and classifies the shadows caused by US probe non-contact that occur on the right and left sides of the US image using the original weights learned. The criteria for shadows were defined as shadows occurring at the left or right edges of the US image and extending continuously from top to bottom within the US image. The rotation is performed around the in-plane lateral axis (Y-axis) of the imaging plane, such that a positive rotation lifts the right side of the probe and a negative rotation lifts the left side. This rotation is applied in the direction that is expected to eliminate the shadows. The adjustment is repeated iteratively until the shadows are sufficiently reduced. When the shadows occur at one scan position on the scan path, they often occur at the next scan position as well. To cancel shadows efficiently, the amount of posture angle corrected at the current scan position is carried over to the posture control at the next scan position (see Fig. 5). The total rotation angle at scan index *i*, denoted $\:{\theta\:}_{Y}\left(i\right)$ is composed of two terms: (1) the pre-planned angle $\:{\theta\:}_{planned}\left(i\right)$ which is estimated with the normal vector on the body surface; (2) the correction term $\:{\theta\:}_{correct}\left(j\right)$ which is updated at each feedback iteration *j* based on the detected presence or absence of shadows. The value of $\:{\theta\:}_{add}\left(j\right)$ varies with the presence or absence of the shadow, and this sum is $\:{\theta\:}_{correct}\left(i\right)$. These processes are expressed in the following equation.

$$\theta _{Y} \left( i \right) = \sum\limits_{{k = 1}}^{{i - 1}} {\theta _{{correct}} \left( k \right) + \theta _{{planned}} \left( i \right) + \theta _{{correct}} \left( i \right)}$$

$$\theta _{{correct}} \left( i \right) = \sum\limits_{{j = 1}}^{M} {\theta _{{add}} \left( j \right) \cdot 1_{{\left\{ {C\left( j \right)} \right\}}} }$$

$$\theta _{{add}} \left( j \right) = \left\{ {\begin{array}{*{20}c} {4.5\quad if\:left\:shadow} \\ { - 4.5\quad if\:right\:shadow} \\ {0\quad else} \\ \end{array} } \right.$$

4 $${C\left( j \right) = ~\left[ {\theta _{{add}} \left( j \right) = 0} \right]}$$

$\:C\left(j\right)$ is an indicator function, which returns 1 if the condition $\:C\left(j\right)$ is satisfied and 0 if the condition is not satisfied. *M* represents the maximum iterations allowed in each step. By integrating all the above components (scan path planning, US probe posture control, and visual servo angle correction), the automated gallbladder detection is realized.

### Experimental setup

In this study, three experiments are performed to evaluate the efficacy of the proposed method for automating the gallbladder imaging under the right subcostal boundary. The first experiment compares the manually planned path with the auto generated path by the proposed estimation method for the right subcostal boundary. The evaluation was based on the detection rate of the gallbladder and the incidence of shadows. The second experiment was performed to validate the proposed US probe posture control and visual servoing, comparing the gallbladder detection rate and the incidence of shadows with and without the application of each control. The third experiment was performed to validate the comprehensiveness of the gallbladder. With the obtained US images in the second experiments, we performed a scoring of the coverage by a physician who is expert in abdominal US. The number of gallbladder necks, bodies, and bottoms included in the US images obtained from a single scan was scored (e.g. 3 points when all parts are included; 2 points when only neck and body are included). Please refer to Supplementary Video 1 for the experiment overview. Those experiments were approved by the institutional review board at National Institute of Advanced Industrial Science and Technology (No. 2024 − 0563) and performed in accordance with relevant guidelines. The informed consent including the publication of the information/image in the online journal was obtained from all participants.

The common conditions for all experiments are as follows.

- Six healthy subjects participated in the experiments. Detailed information is listed in Table 1. Note that those subjects are not included in the training data of the rib region detection and the shadow detection.
- Since the gallbladder expels bile after meal ingestion and is less visible on US images^30^, the subjects participated in the experiment after having not eaten for at least 6 h.
- The subjects lay on the bed in the supine position.
- The experiment was started with US gel applied to the body surface in advance.
- For longitudinal scanning along the right subcostal boundary, the probe’s $\:{\theta\:}_{Z}$ rotation was fixed to be parallel to the body axis direction.
- The force feedback control was applied so that the contact force in the vertical axis of the US probe was maintained at 5 N as accepted in the related paper^8^.
- Before the scanning starts, the subjects are instructed to take a deep breath and hold their breath.
- The gallbladder detection rate is defined as the percentage of the number of trials in which the gallbladder is depicted in the US image out of all trials.
- The incidence of shadows is defined as the percentage of the number of US images in which shadow occurred out of the number of all images obtained in each trial. The shadow incidence count was performed using automatically detected shadows by YOLOv8.

**Table 1:** Subject profile.

No.	Height [cm].	Weight [kg].	BMI	Age	Gender
1	180	71	21.9	24	Male
2	168	59	20.9	23	Male
3	169	69	24.2	34	Male
4	173	69	23.1	24	Male
5	168	68	24.1	23	Male
6	177	54	17.2	24	Male

### Path planning validation

In this experiment, scanning paths generated by the estimation method using rib detection were compared with scanning paths manually planned by human. The RGB-D camera was placed roughly 700 mm above the bed so that the upper body was within the camera’s imaging range. The manual scanning path was created by the physician viewing the RGB image sent from the RGB-D camera on the monitor and tracing over the RGB image with the mouse cursor. The following procedure was used to create the automatically generated scanning path using the proposed method. First, the subject was instructed to take a deep breath by voice guidance from the system, and the RGB-D image was captured and the depth difference image was generated in accordance with the timing of the breath. Next, the subcostal boundary estimation using YOLOv5 was performed on the generated depth difference image. The weights for this estimation model were trained using 581 training data collected from 9 subjects with BMI 18.8–42.0 in advance. An automatically generated path is defined as a path offset to the estimated line by 30 mm to the leg side, which is half of the total length of the imaging plane of the US probe. The same subject was scanned manually and by the proposed method, with the posture control applied to each path, and the success rate of gallbladder detection and the incidence of shadows were compared. A total of 30 scans were performed on the subjects in each the condition.

### Probe control validation

In this experiment, we compared the results with and without the posture control and with and without the shadow cancellation control by visual servoing. For the scanning paths, the automatically generated paths by the proposed method are used. When the posture control of the US probe is not applied, the rotational angles $\:{\theta\:}_{X}$ and $\:{\theta\:}_{Y}$were fixed at 0 in the robot coordinate system. In the visual servoing, to classify the left and right shadows, we used YOLOv8 trained by 656 US images collected from one subject in advance. If the shadows were detected, Eq. (4) of $\:{\theta\:}_{add}$ was updated and the angle was corrected in 4.5 degrees steps. The frequency of the feedback control loop was 30 Hz. Comparisons were made regarding the gallbladder detection rate and the incidence of the shadows. A total of 60 scans were performed on the subjects in each the condition.

## Results

### Path planning

Of all scans, the gallbladder was seen in 86.7% of the manual scan paths and 83.3% of the automatically generated scan paths, as shown in Table 2. There was no difference in the appearance of structures other than the gallbladder in the US images between the manual and the automatically generated scan paths. The incidence of shadows is also shown in Table 2. A one-tailed t-test between the manual and automatically generated scanning paths showed no statistically significant difference, *p* = 0.29. It was shown that the estimated scanning path based on the position of the right subcostal boundary was not inferior to the manually planned one in terms of both gallbladder imaging and shadow incidence.

**Table 2:** Each experimental scenario and results of detection rate of gallbladder and shadow.

	Path planning	US probe control		Detection rate [%]	
		Posture control	Visual servoing	Gallbladder	Shadow
Exp. 1	No	Yes	No	86.7	14.3 ± 12.9
	Yes	Yes	No	83.3	16.6 ± 14.9
Exp. 2	Yes	No	No	58.3	53.4 ± 24.6
	Yes	Yes	No	81.7	17.7 ± 18.4
	Yes	No	Yes	65.0	24.6 ± 18.4
	Yes	Yes	Yes	78.3	12.6 ± 15.5

### Probe control

**Fig. 6:**
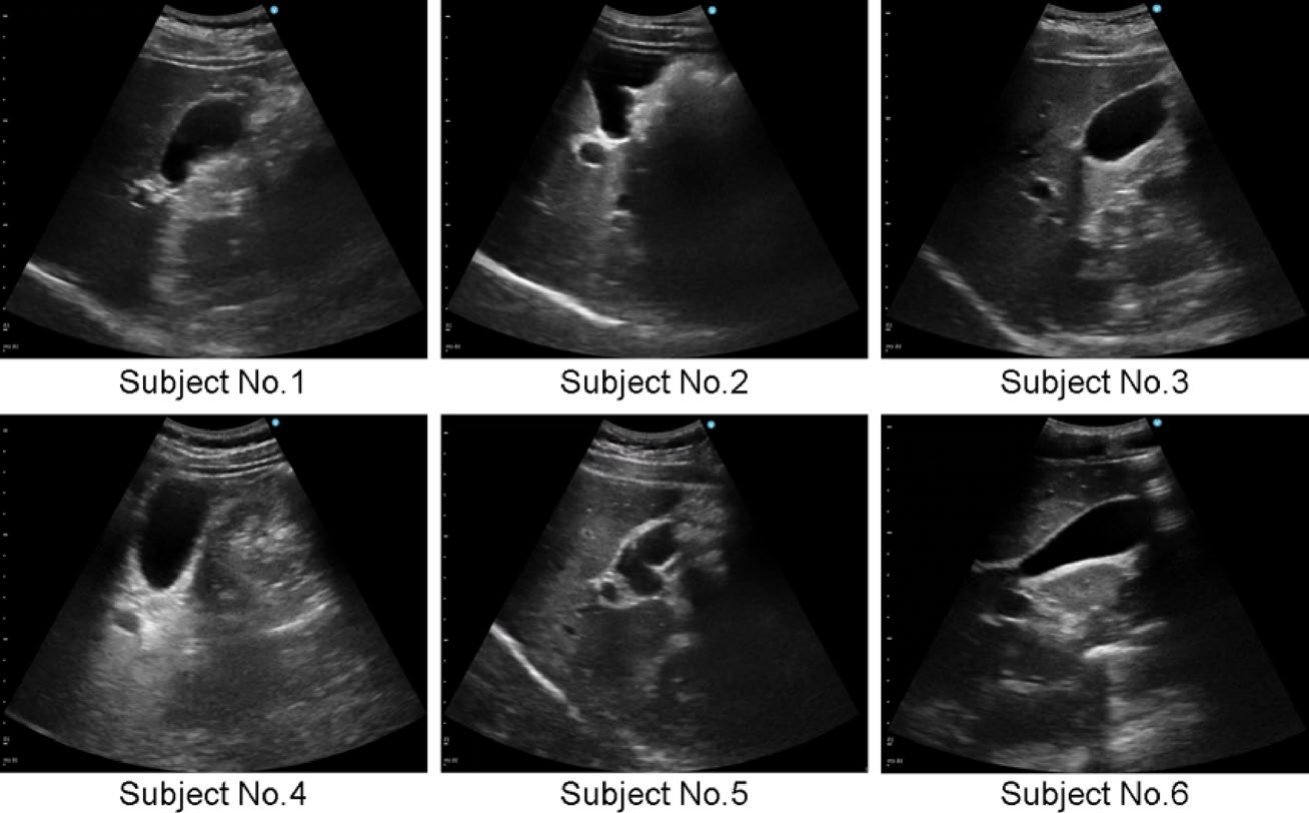
Acquired US images showing the gallbladder of each subject.

The gallbladder detection rates were 58.3% when neither posture control (PC) nor visual servoing (VS) was applied, 81.7% with PC alone, 65.0% with VS alone, and 78.3% when both PC and VS were applied (see Table 2). The detection of gallbladder was found to be significantly different with and without PC. The incidence of shadows was the lowest when VS was applied, at an average of 12.6% (see Table 2). A one-tailed t-test of the shadow incidence rate with and without VS showed a statistically significant difference of *p* = 0.001. The mean scanning times were 23.3 ± 2.2 s without PC or VS, 24.1 ± 3.1 s with PC alone, 30.4 ± 2.8 s with VS alone, and 27.5 ± 4.2 s when both PC and VS were applied. Representative examples of the US images of the gallbladder detected in each subject are shown in Fig. 6. The US image of the gallbladder in the subject No. 5 showed polyps in the gallbladder.

### Gallbladder coverage

The score of the gallbladder coverage was 1.23 ± 0.99 when PC and VS were not applied, 1.68 ± 1.07 when PC was applied and VS was not applied, 1.47 ± 0.89 when PC was not applied and VS was applied, and 1.63 ± 1.05 when both PC and VS were applied. There was a significant difference between the condition in which PC and VS were not applied, and the conditions in which only PC was applied and both PC and VS were applied (*p* < 0.01). Focusing on the results per subject, the scores for No. 1 and 2 were relatively high, while the score for No. 3, who had the relatively high BMI, was notably low.

## Discussion

When the posture control was applied, the detection rate of the gallbladder improved. One possible explanation is related to the geometry of the body surface near the subcostal region. In individuals with a standard body type, the height of the abdomen is often lower than the height of the chest in the supine position (see Fig. 4), meaning the body surface may slope downward from the chest toward the abdomen. Under such conditions, placing the US probe perpendicular to the body surface may naturally orient it in a direction that “looks up” toward the underside of the rib. Since the gallbladder is often located beneath or partially obscured by the rib cage, this orientation could contribute to improved visualization. In contrast, in individuals with higher BMI, the slope of the body surface may differ from that of the standard body type. As a result, posture control based on the local surface normal could rotate the probe in a direction that does not favor gallbladder visualization. This may help explain why the gallbladder was difficult to detect in the trials of subject No. 3, who had a relatively high BMI; however, we cannot conclude BMI as the sole cause, and additional anthropometric factors may also be involved. To accommodate a wide range of body types, it may be necessary to develop a method that adjusts the probe posture to “look up” toward the underside of the ribs—such as a gallbladder-centered visual servoing approach—rather than relying strictly on the local surface normal. Furthermore, although the contact force in our experiments was fixed at 5 N, patients with higher BMI may require greater force for adequate visualization; this is another factor that may have contributed to the observed variability.

Although the lowest shadow incidence would be expected when both posture control and visual servoing are applied, there was one trial in which the shadow incidence rate was unexpectedly high (72%). In this trial, shadows appeared simultaneously on both the left and right sides of the US image. Typically, when bilateral shadows are caused by poor contact, they can be reduced simply by increasing the probe’s contact force. However, in this particular case, the left-side shadow was caused by insufficient contact, while the right-side shadow resulted from rib interference. This combination occurred because the scan path was positioned too close to the ribs, making it difficult for the system to correct both issues simultaneously. It should also be noted that the severity of detected shadows was not precisely differentiated, as the criteria in this study simply defined shadows as those occurring at the image edges and extending continuously from top to bottom. Future work may benefit from distinguishing between mild shadows (e.g., due to insufficient contact) and more severe rib shadows, and incorporating this information into robotic control. It may also be useful to allow the scan path to adjust dynamically when unanticipated shadows are encountered.

When visual servoing was applied, the detection rate of the gallbladder and the coverage score were slightly lower than when it was not applied. One possible interpretation is that the probe’s rotation to reduce shadowing may have caused the gallbladder to move temporarily out of plane. At the same time, factors such as the subject’s breathing pattern, natural anatomical variation, and the accuracy of scan path planning may also influence detection. Given that the reduction was small, it is difficult to attribute this effect primarily to the visual servoing.

Breathing variability presents another source of uncertainty. Subjects were instructed to take a deep breath and hold it, as in clinical practice^31^, but the depth and mode of breathing (abdominal vs. thoracic) differed slightly among trials. Such differences may influence both posture control based on the body surface normal and the actual gallbladder position. Additionally, discrepancies between breathing during path generation and during scanning may cause mismatches between the planned and executed scan positions. Addressing this may require classifying or quantifying subjects’ breathing patterns and adapting scan paths accordingly, or offering additional instructions for suboptimal breathing. Since the scan path in this study was generated before robot motion began, real-time updates to the path based on ongoing observations of the body surface could help reduce these mismatches. Furthermore, if the gallbladder is difficult to detect from the right subcostal approach, alternative paths—such as intercostal scanning—may be necessary.

Finally, challenges remain in achieving full gallbladder coverage. In this study, only one scan was performed along the rib boundary based on the planned path. To obtain more comprehensive gallbladder imaging, it may be beneficial to rotate the probe so that the gallbladder remains centered relative to the estimated path, or to incorporate additional sweep strategies during scanning.

## Conclusion

The aim of this study was to automate gallbladder detection in abdominal ultrasonography. We developed a scanning path planning method based on rib region detection, along with a probe control approach incorporating posture control and shadow-avoiding visual servoing. Human trials with six subjects demonstrated the feasibility of automated gallbladder detection using the proposed methods. The sample in this study—six healthy young male volunteers with BMIs between 17.2 and 24.2—constitutes a limited cohort, and the absence of overweight or obese participants restricts the broader applicability of the results. Further studies involving larger and more diverse populations will be necessary to validate the feasibility of the proposed methods. We would also like to expand the scope of application to automated imaging of abdominal organs other than the gallbladder such as liver and kidneys.

## Supplementary Information

Below is the link to the electronic supplementary material.

Supplementary Material 1

Supplementary Material 2

## Data Availability

The data set used in this study is available from the corresponding author upon reasonable request.

## References

[1] Shung, K. Diagnostic ultrasound: Past, present, and future. *J. Med. Biol. Eng.* **31**, 371–374 (2011).

[2] Berg, W. A., Blume, J. D., Cormack, J. B. & Mendelson, E. B. Operator dependence of physician-performed whole-breast US: Lesion detection and characterization. *Radiology* **241**, 355–365 (2006). 17057064 10.1148/radiol.2412051710

[3] Zhang, D. & Huang, H. Prevalence of work-related musculoskeletal disorders among sonographers in china: Results from a National web-based survey. *J. Occup. Health* **59**, 529–541. 10.1539/joh.17-0057-OA (2017). 28904258 10.1539/joh.17-0057-OA PMC5721275

[4] Sweeney, K., O’Connor, C., Hewson, D. & Hellyer, R. The effectiveness of ergonomics interventions in reducing upper limb work-related musculoskeletal pain and dysfunction in sonographers, surgeons and dentists: A systematic review. *Ergonomics* **64**, 1–38 (2020). 32866082 10.1080/00140139.2020.1811401

[5] Li, K., Xu, Y. & Meng, M. Q. H. An overview of systems and techniques for autonomous robotic ultrasound acquisitions. *IEEE Trans. Med. Robot Bionics*. **3**, 510–524 (2021).

[6] Noone, T. C., Semelka, R. C., Chaney, D. M. & Reinhold, C. Abdominal imaging studies: Comparison of diagnostic accuracies resulting from ultrasound, computed tomography, and magnetic resonance imaging. *Magn. Reson. Imaging* **22**, 19–24 (2004). 14972390 10.1016/j.mri.2003.01.001

[7] Mustafa, A. S. B., Alhussein, O., Babiker, M. & Mathkour, H. Development of a robotic system for autonomous liver screening using ultrasound scanning device. In *Proc. IEEE Int. Conf. Robot. Biomimetics (ROBIO)* 804–809 (2013).

[8] Virga, S., Mahmood, F., Mercaldo, N., Dagnino, R. & Navab, N. Automatic force-compliant robotic ultrasound screening of abdominal aortic aneurysms. In *Proc. IEEE/RSJ Int. Conf. Intell. Robots Syst. (IROS)* 508–513 (2016).

[9] Adams, R. B. Ultrasound scanning techniques. *Surg. Open. Sci.* **10**, 182–207 (2022). 36324368 10.1016/j.sopen.2022.09.002 PMC9619033

[10] Mustafa, A. S. B. et al. Human abdomen recognition using camera and force sensor in medical robot system for automatic ultrasound scan. In *Proc. IEEE Eng. Med. Biol. Soc.* 4855–4858 (2013). 10.1109/EMBC.2013.6610635 24110822

[11] Victorova, M., Lee, M. K. S., Navarro-Alarcon, D. & Zheng, Y. Follow the curve: Robotic ultrasound navigation with learning-based localization of spinous processes for scoliosis assessment. *IEEE Access.* **10**, 40216–40229 (2022).

[12] Shida, Y., Kumagai, S., Tsumura, R. & Iwata, H. Automated image acquisition of parasternal long-axis view with robotic echocardiography. *IEEE Robot Autom. Lett.* **8**, 5228–5235 (2023).

[13] Ma, X., Zhang, Z. & Zhang, H. K. Autonomous scanning target localization for robotic lung ultrasound imaging. In *Proc. IEEE/RSJ Int. Conf. Intell. Robots Syst. (IROS)* 9467–9474 (2021). 10.1109/iros51168.2021.9635902 PMC9373068 35965637

[14] Tsumura, R., Koseki, Y., Nitta, N. & Yoshinaka, K. Towards a fully automated robotic platform for remote auscultation. *Int. J. Med. Robot Comput. Assist. Surg.* **19**, e2461 (2023). 10.1002/rcs.2461 36097703

[15] Tan, J., Wang, W., Yang, J. & Li, X. Fully automatic dual-probe lung ultrasound scanning robot for screening triage. *IEEE Trans. Ultrason. Ferroelectr. Freq. Control*. **70**, 975–988 (2023). 36191095 10.1109/TUFFC.2022.3211532

[16] Deng, X., Chen, Y., Chen, F. & Li, M. Learning robotic ultrasound scanning skills via human demonstrations and guided explorations. In *Proc. IEEE Int. Conf. Robot. Biomimetics (ROBIO)* 372–378 (2021).

[17] Bi, Y., Wu, J., Chen, H. & Li, Y. Autonomous path planning for intercostal robotic ultrasound imaging using reinforcement learning. *ArXiv Preprint arXiv* :240409927 (2024). 10.1038/s41598-026-37702-9 PMC12905323 41673226

[18] Huang, Q., Lan, J. & Li, X. Robotic arm based automatic ultrasound scanning for three-dimensional imaging. *IEEE Trans. Ind. Inf.* **15**, 1173–1182 (2019).

[19] Kojcev, R., Khakzar, A., Fuerst, B., Navab, N. & Wein, W. On the reproducibility of expert-operated and robotic ultrasound acquisitions. *Int. J. Comput. Assist. Radiol. Surg.* **12**, 1003–1011 (2017). 28321804 10.1007/s11548-017-1561-1

[20] Ma, X., Kuo, W. Y., Yang, K., Rahaman, A. & Zhang, H. K. A-SEE: Active-sensing end-effector enabled probe self-normal-positioning for robotic ultrasound imaging applications. *IEEE Robot Autom. Lett.* **7**, 12475–12482 (2022). 37325198 10.1109/lra.2022.3218183 PMC10266708

[21] Bao, X., Wang, Y., Li, Z. & Zhang, F. SAPM: Self-adaptive parallel manipulator with pose and force adjustment for robotic ultrasonography. *IEEE Trans. Ind. Electron.* **70**, 10333–10343 (2023). 37323755 10.1109/TIE.2022.3220864 PMC7614654

[22] Jiang, Z., Huang, Q., Li, X. & Liu, H. Automatic normal positioning of robotic ultrasound probe based only on confidence map optimization and force measurement. *IEEE Robot Autom. Lett.* **5**, 1342–1349 (2020).

[23] Okuzaki, K., Koizumi, N., Yoshinaka, K. & Nishiyama, Y. Rib region detection for scanning path planning for fully automated robotic abdominal ultrasonography. *Int. J. Comput. Assist. Radiol. Surg.* **19**, 449–457 (2024). 37787939 10.1007/s11548-023-03019-5

[24] Tsumura, R., Koseki, Y., Nitta, N. & Yoshinaka, K. Safe contact force generation for robotic thyroid ultrasound imaging. *IEEE Robot Autom. Lett.* **9**, 1700–1707 (2024).

[25] Al-Zogbi, L., Al-Darraji, H., Hussein, A. & Salman, A. Autonomous robotic point-of-care ultrasound imaging for monitoring of COVID-19-induced pulmonary diseases. *Front. Robot AI*. **25**, 645756 (2021). 10.3389/frobt.2021.645756 PMC8185340 34113656

[26] Mustafa, A. S. B. et al. Human abdomen recognition using camera and force sensor in medical robot system for automatic ultrasound scan. *In Proc. IEEE Eng. Med. Biol. Soc.*, 4855–4858 (2013). 10.1109/EMBC.2013.6610635 24110822

[27] Jocher, G. et al. YOLOv5. (2020). https://github.com/ultralytics/yolov5

[28] Katsuragi, A., Nakamura, Y., Takahashi, S. & Saito, K. Detection of organs and classification of missing parts using deep learning for automated ultrasound diagnosis. In *Proc. 34th Annu. Meeting Jpn. Soc. Ultrasound Med. Kanto Koshinetsu Region* 107 (2022).

[29] Jocher, G. et al. YOLOv8. (2023). https://github.com/ultralytics/ultralytics

[30] Ehrenstein, B., Froh, S., Schlottmann, K., Schölmerich, J. & Schacherer, D. Effect of fasting prior to abdominal sonography examinations on the quality of imaging under routine conditions: A randomized, examiner-blinded trial. *Scand. J. Gastroenterol.* **44**, 1048–1054 (2009). 19562622 10.1080/00365520903075188

[31] Weill, F. S. The bile ducts: Examination techniques and sonoanatomy. *Ultrasound Diagnosis Dig. Diseases* (1990).

