# Supplementary material for: Fully automated robotic ultrasound gallbladder imaging with subcostal scanning
Source: Sci Rep. 2025 Dec 10;16:2084. doi: 10.1038/s41598-025-31892-4 (PMC12808744; doi:10.1038/s41598-025-31892-4)
Supplement: Supplementary file 2 — Supplementary Material 2 [file 41598_2025_31892_MOESM2_ESM.pdf]

Description of Additional Supplementary File

Supplementary Video 1: Demonstration of fully automated gallbladder scanning using the proposed path planning and control method for robotic ultrasound
